# *NKX2-3* Transcriptional Regulation of Endothelin-1 and VEGF Signaling in Human Intestinal Microvascular Endothelial Cells

**DOI:** 10.1371/journal.pone.0020454

**Published:** 2011-05-26

**Authors:** Wei Yu, John P. Hegarty, Arthur Berg, Xi Chen, Gail West, Ashley A. Kelly, Yunhua Wang, Lisa S. Poritz, Walter A. Koltun, Zhenwu Lin

**Affiliations:** 1 Department of Surgery, Pennsylvania State University, Hershey, Pennsylvania, United States of America; 2 Center for Statistical Genetics, Department of Public Health Sciences, Pennsylvania State University, Hershey, Pennsylvania, United States of America; 3 Department of Cellular & Molecular Physiology, Pennsylvania State University, Hershey, Pennsylvania, United States of America; 4 Department of Biostatistics, Vanderbilt University, Nashville, Tennessee, United States of America; 5 Department of Pathobiology, Lerner Research Institute, the Cleveland Clinic Foundation, Cleveland, Ohio, United States of America; Kyushu Institute of Technology, Japan

## Abstract

**Background:**

*NKX2-3* is associated with inflammatory bowel disease (IBD). *NKX2-3* is expressed in microvascular endothelial cells and the muscularis mucosa of the gastrointestinal tract. Human intestinal microvascular endothelial cells (HIMECs) are actively involved in the pathogenesis of IBD and IBD-associated microvascular dysfunction. To understand the cellular function of *NKX2-3* and its potential role underlying IBD pathogenesis, we investigated the genes regulated by *NKX2-3* in HIMEC using cDNA microarray.

**Methodology/Principal Findings:**

*NKX2-3* expression was suppressed by shRNA in two HIMEC lines and gene expression was profiled by cDNA microarray. Pathway Analysis was used to identify gene networks according to biological functions and associated pathways. Validation of microarray and genes expression in intestinal tissues was assessed by RT-PCR. *NKX2-3* regulated genes are involved in immune and inflammatory response, cell proliferation and growth, metabolic process, and angiogenesis. Several inflammation and angiogenesis related signaling pathways that play important roles in IBD were regulated by *NKX2-3*, including endothelin-1 and VEGF-PI3K/AKT-eNOS. Expression levels of *NKX2-3*, *VEGFA*, *PI3K*, *AKT*, and *eNOS* are increased in intestinal tissues from IBD patients and expression levels of *EDN1* are decreased in intestinal tissues from IBD patients. These results demonstrated the important roles of *NKX2-3*, *VEGF*, *PI3K*, *AKT*, *eNOS*, and *EDN1* in IBD pathogenesis. Correlation analysis showed a positive correlation between mRNA expression of *NKX2-3* and *VEGFA* and a negative correlation between mRNA expression of *NKX2-3* and *EDN1* in intestinal tissues from IBD patients.

**Conclusion/Relevance:**

*NKX2-3* may play an important role in IBD pathogenesis by regulating endothelin-1 and VEGF signaling in HIMECs.

## Introduction

Crohn's disease (CD) and ulcerative colitis (UC), the two main subtypes of inflammatory bowel disease (IBD), are chronic, relapsing inflammatory disorders of the gastrointestinal tract. Genetics play an important role in the development of IBD [1]. *NKX2-3* (NK2 transcription factor related, locus 3) has been shown to be associated with both CD and UC by recent genome-wide association studies [2], [3].

*NKX2-3* is a member of the Nkx family of homeodomain transcription factors that play critical roles in regulating tissue-specific gene expression essential for determining tissue differentiation, as well as the temporal and spatial patterns of development [4], [5]. During development, *NKX2-3* is primarily expressed in the midgut and hindgut mesoderm and spleen, as well as in pharyngeal endoderm [6], [7], [8]. Analysis of *NKX2-3*-deficient mice has revealed a critical role for this homeobox transcription factor in spleen development and organization [9], and in establishing the correct environment for normal B cell development and T cell dependent immune response [10]. *NKX2-3* is also essential for normal small intestine development and function [11]. *NKX2-3* is also expressed in microvascular endothelial cells within the lamina propria and submucosa of the intestine, where it is required for expression of the lymphocyte adhesion molecule MAdCAM-1 in the mouse [9].

Microvascular endothelial cells have a critical “gatekeeper” role in the inflammatory process through their ability to recruit circulating immune cells to foci of inflammation. Endothelial activation in response to cytokines and bacterial products results in cell adhesion molecule expression and chemokine production, which mediate increased binding and transmigration of leukocytes across the vascular wall. Intestinal microvascular endothelial cells are recognized as a cell population actively involved in the pathogenesis of inflammatory bowel diseases (IBD) and IBD-associated microvascular dysfunction [12].

Transcription factors can regulate the expression of downstream genes. Recently, we found that the expression of *NKX2-3* is up-regulated in intestinal tissues and B cells from CD patients [13] and subsequently identified many inflammation and immune-response genes regulated by *NKX2-3* in B cell lines from a CD patient. These included several genes which also have important functions in endothelial cells, such as endothelin-1 (*EDN1*), *ASS1, and KLF2* [14]. Since endothelial cells play a key role in mucosal immune homeostasis and *NKX2-3* is expressed in intestinal endothelial cells, we further performed cDNA microarray to identify genes regulated by *NKX2-3* in two human intestinal microvascular endothelial cell lines (HIMEC).

## Results

### Suppression of *NKX2-3* expression in 2 HIMEC lines

**Figure 1:**
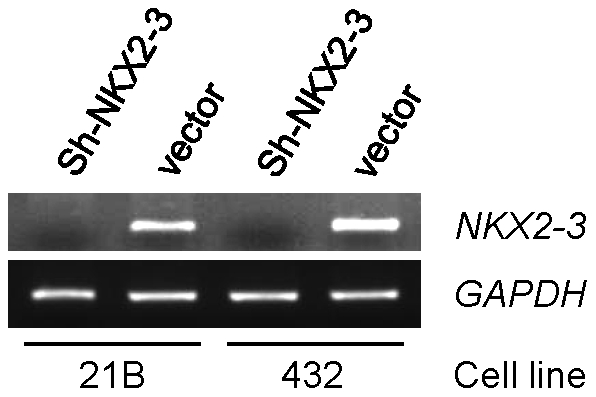
*NKX2-3* mRNA expression is suppressed by shRNA in 21B and 432 HIMEC. pSUPER.retro.puro.shRNA-*NKX2-3* and empty vector were transfected into two HIMEC. 48 hours after transfection, RNA was isolated and analyzed by RT-PCR in two knockdown HIMEC compared with controls. GAPDH expression served as a control.

pSUPER.retro.puro.shRNA-*NKX2-3* and empty vector were transfected into 21B and 432 HIMEC. RT-PCR results showed that mRNA expression levels of *NKX2-3* in the two shRNA-*NKX2-3* cells were significantly reduced compared with the empty vector cells (control cells) 48 hours after transfection (Fig. 1).

### Identification of genes regulated by *NKX2-3*

To analyze the effects of *NKX2-3* knockdown on gene expression and identify genes with altered expression levels, cDNA microarray analysis was conducted with *NKX2-3* knockdown and control cells from two HIMEC. Stringent criteria (fold change ≥1.5, up or down, *p*<0.0005) were used to filter the differentially expressed genes. The expression levels of 1746 genes were affected by *NKX2-3* knockdown (935 down-regulated and 811 up-regulated) in the HIMEC 21B cell line as compared to control, and 1603 genes were affected by *NKX2-3* knockdown (741 down-regulated and 862 up-regulated) in the HIMEC 432 cell line as compared to control. A total of 1000 shared genes were found to be affected by *NKX2-3* knockdown in both HIMEC, including 996 (99.6%) genes in the same direction (432 genes down-regulated and 564 genes up-regulated by *NKX2-3* knockdown in both HIMEC), and only 4 genes in the opposite direction. Taken together, the transcriptional profile of genes affected by *NKX2-3* knockdown was highly consistent for both HIMEC. Table 1 shows the top 100 down-regulated and top 100 up-regulated genes by *NKX2-3* knockdown with average fold changes in the two cell lines. In order to characterize the top genes affected by *NKX2-3* knockdown, they were assigned to ontological functional groups based on IPA and references in the literature. These 200 genes grouped primarily within the following functional categories, which are listed as such in Table 1: immune and inflammatory response; cell growth and proliferation; metabolic process; cell adhesion; transcription regulation; transport and structure; and angiogenesis. Since the *NKX2-3* transcription factor is associated with IBD and is up-regulated in CD patients [13], it is reasonable to assume that *NKX2-3* could play a role in IBD pathogenesis by regulating inflammation-related genes. In fact, among the 200 genes regulated by *NKX2-3*, 70 genes were immune and inflammatory response genes, including *IL8* (array ratio 0.4) and *KLF2* (array ratio 3.92). Angiogenesis plays an important role in endothelial cell participation in inflammation [15], *ANGPT2* is down-regulated (array ratio 0.18) and *FGF2* is up-regulated (array ratio 4.0) by *NKX2-3* knockdown.

**Table 1:** Top 200 genes regulated by NKX2-3 knockdown in HIMEC.

Gene	Fold	*p*-value	Gene	Fold	*p*-value	Gene	Fold	*p*-value	Gene	Fold	*p*-value
***Cell growth and proliferation***			***Transport and structure***			***Inflammation and immune response***			***Inflammation and immune response***		
PIM3	0.3	2.31E-06	SYT11	0.35	3.66E-07	TNFRSF10D	0.31	3.54E-07	SAMD9	7.31	2.01E-10
TFPI2	0.32	3.94E-06	KRT81	0.4	3.31E-07	TNFSF18	0.32	6.43E-05	STAT1	7.72	1.98E-09
NR2F1	0.37	4.64E-07	RPS23	0.4	2.97E-07	CERK	0.33	1.39E-06	TAP2	7.83	7.33E-10
EIF4B	0.38	2.68E-07	EXOC6	0.47	2.09E-06	FAM172A	0.37	1.94E-07	PSMB8	8.22	1.84E-10
GHR	0.39	3.69E-07	RPL23	0.47	1.33E-06	YPEL2	0.39	0.000952	UBE2L6	8.33	9.08E-10
HMMR	0.39	3.79E-06	RPS29	0.48	2.41E-06	PAPSS2	0.39	8.84E-06	PTGS2	8.63	2.77E-11
REEP1	0.4	2.86E-07	ADAP1	3.29	2.36E-08	IL8	0.4	7.63E-06	GBP1	9.24	2.23E-08
ZMAT3	0.41	3.60E-07	SLC25A28	3.71	1.99E-07	UACA	0.4	3.05E-05	PARP9	9.46	6.09E-10
MMP7	0.42	3.43E-06	RTP4	6.47	0.000111	GIMAP7	0.41	9.24E-07	SP110	9.83	1.31E-10
GDF15	0.42	1.22E-05	GJD3	7.95	0.000154	HES2	0.41	2.05E-07	TAP1	10.62	7.18E-11
MFNG	0.42	2.32E-05				SLC40A1	0.41	2.59E-07	USP18	11.2	9.63E-11
AGAP3	0.43	6.31E-07				TNFAIP8L1	0.43	3.66E-07	DDX58	11.39	3.09E-08
TMEM158	0.44	1.25E-05	***Cell adhesion***			F2RL1	0.43	0.000121	HLA-B	11.89	8.66E-12
UNC5A	0.44	7.44E-05				CD36	0.43	5.14E-07	PRIC285	12.35	1.67E-10
SHMT2	0.44	1.55E-06	POSTN	0.22	6.28E-09	PLD1	0.44	2.12E-07	PSMB9	14.18	1.73E-10
FAM198B	0.45	3.44E-06	MMRN1	0.35	3.33E-06	CBS	0.46	3.70E-06			
HNRNPA0	0.46	6.11E-05	CXADR	0.36	7.75E-07	CEP55	0.47	3.69E-06			
PAWR	0.46	4.71E-05	SRPX	0.37	7.57E-06	GNG12	0.48	5.21E-05	***Others***		
DCLK1	0.47	7.04E-07	FLRT2	0.46	8.02E-06	TNFSF4	0.48	1.00E-05			
TTC3	0.47	5.53E-07	AIF1L	3.41	8.77E-08	ARHGDIB	0.49	2.55E-05	ANKRD55	0.18	1.07E-07
TFF3	0.47	1.73E-05				CLEC1A	0.49	1.96E-05	C2CD4B	0.22	2.48E-08
PTPRE	0.47	4.31E-06				CXCL16	3.19	1.04E-05	KIAA0114	0.27	2.03E-08
UBE4B	0.47	2.02E-06	***Transcription regulation***			GCH1	3.32	5.31E-05	NT5DC2	0.28	8.23E-09
CDKN3	0.48	0.000101				UNC93B1	3.46	1.36E-06	EIF4BP7	0.29	3.73E-07
CCNB2	0.49	2.92E-05	SDPR	0.44	1.95E-06	CD68	3.51	3.49E-07	FLJ41200	0.29	2.64E-08
ANLN	0.49	3.85E-05	HOXD1	0.45	0.000147	TRIM21	3.62	9.21E-06	C13orf33	0.34	7.43E-06
LGMN	3.23	4.42E-07	ZNFX1	3.52	2.22E-06	APOBEC3G	3.67	5.26E-06	EIF4BP3	0.38	1.42E-07
SEMA3A	3.25	2.19E-06	BATF2	4.11	1.75E-06	TRIM5	3.82	4.78E-05	FBN2	0.39	5.49E-06
PNPT1	3.44	2.45E-05				KLF2	3.92	7.66E-06	C7orf41	0.4	5.68E-07
MAP2	3.57	4.49E-09				PLSCR1	4.05	2.58E-08	SNHG9	0.41	0.000156
PMAIP1	3.65	3.66E-08	***Metabolic process***			MYD88	4.28	1.98E-06	WRB	0.42	1.18E-06
EIF2AK2	3.73	9.30E-09				CD38	4.28	6.55E-08	BCYRN1	0.42	0.000157
HEY1	3.85	2.33E-07	ACO1	0.2	8.00E-07	CX3CL1	4.33	0.000209	ZNF704	0.44	5.90E-07
CSRNP1	3.87	1.55E-06	HSD17B2	0.23	2.48E-06	STAT2	4.34	1.08E-05	ANKRD37	0.46	5.41E-06
C8orf4	3.95	5.53E-06	PHGDH	0.29	1.86E-08	PLEKHA4	4.37	1.83E-05	HNRPA1L-2	0.46	8.27E-06
XAF1	4.17	2.88E-06	PSAT1	0.3	1.24E-07	IFNB1	4.37	3.68E-06	TCTEX1D2	0.47	0.000153
SERPINE2	4.33	2.29E-08	ASNS	0.31	1.09E-07	FCN3	4.38	0.000198	CENPW	0.48	7.38E-05
HEY2	4.6	4.55E-08	GALNTL2	0.33	7.00E-06	F2RL3	4.44	2.45E-07	NETO2	0.48	1.93E-05
PARP10	5.54	5.42E-08	PKIA	0.34	7.05E-05	ZC3HAV1	4.6	5.95E-07	C8orf45	0.48	8.75E-05
CRYAB	6.8	8.25E-06	MYO5A	0.38	5.34E-05	HCG4	4.61	2.85E-09	MAMDC2	3.19	6.34E-07
KLF4	6.86	6.09E-10	TXNDC12	0.4	1.43E-07	HLA-C	4.63	1.63E-09	FBXO6	3.26	7.21E-08
LY6E	7.47	1.94E-10	SNCAIP	0.41	0.000257	GBP2	4.71	4.44E-08	C1orf74	3.48	0.000186
TMEM100	15.53	3.36E-07	CHST7	0.44	7.79E-07	SMAD7	4.78	1.31E-06	USP41	3.52	3.72E-06
			MCTP1	0.46	0.000231	NT5C3	4.8	1.92E-07	C19orf66	3.92	2.77E-08
			FAR2	0.48	0.000238	HLA-H	5.39	4.83E-10	HIST2H2AC	4.18	5.43E-08
***Angiogenesis***			PRSS12	0.48	1.43E-06	TRIM22	5.49	9.22E-08	GCA	5.14	5.15E-06
			BCHE	0.49	3.81E-06	DHX58	5.58	1.57E-06	HIST2H2AA3	5.17	2.92E-09
ANGPT2	0.18	1.42E-05	ADAMTSL2	3.2	1.26E-06	IL18BP	5.66	3.92E-09	HIST2H2AA4	5.28	2.11E-09
SRPX2	0.33	0.000284	TIPARP	3.28	1.18E-07	HLA-F	5.79	2.68E-10	RNF213	6.6	1.03E-06
TRPC6	0.33	1.08E-07	PLA1A	4.75	3.24E-08	CASP1	5.82	5.35E-10	DDX60L	6.75	1.14E-08
BTG1	0.43	3.66E-05	GMPR	4.8	5.63E-08	PARP12	6.28	1.97E-09	LYPD1	6.75	3.01E-05
BMP4	0.48	2.53E-05	LAP3	6.07	9.20E-09	IDO1	6.93	1.77E-08	DDX60	7.87	2.71E-09
ANGPTL4	3.37	1.01E-08	UBA7	6.64	9.54E-08	HCP5	6.99	3.95E-10	FAM46A	8.63	4.81E-06
TYMP	3.38	1.13E-08	CMPK2	10.3	5.63E-08	PARP14	7.01	1.24E-08	MT1M	8.77	6.76E-11
FGF2	4	5.16E-05	ALPL	12.12	0.000474	IL7R	7.06	3.94E-05	SAMD9L	9.07	1.22E-08

### Identification of pathways regulated by *NKX2-3*

**Figure 2:**
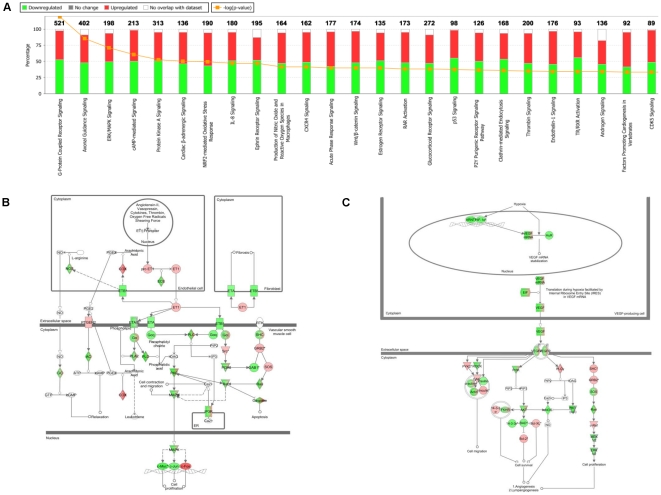
An interaction network generated using IPA analysis shows genes regulated by *NKX2-3* in HIMEC. (A) The top 25 signaling pathways affected by *NKX2-3* knockdown. (B) The EDN1 pathway is regulated by *NKX2-3* in 2 HIMEC. IPA analysis shows genes up-regulated (red) and down-regulated (green) by *NKX2-3* knockdown. (C) The VEGF-PI3K/AKT-eNOS pathway is regulated by *NKX2-3* in 2 HIMEC. IPA analysis shows genes up-regulated (red) and down-regulated (green) by *NKX2-3* knockdown in the VEGF-PI3K/AKT-eNOS pathway.

To assess the global effects of *NKX2-3* on gene expression, IPA was used to systematically visualize the relationships among genes regulated by *NKX2-3* knockdown. The 25 pathways most affected by *NKX2-3* are shown in Fig. 2A. G-Protein Coupled Receptor Signaling (*p* = 1.71×10^−14^), Axonal Guidance Signaling (*p* = 5.80×10^−11^), and ERK/MAPK Signaling (*p* = 5.42×10^−9^) were the top three canonical pathways affected by *NKX2-3* knockdown.

We previously found that knockdown of *NKX2-3* in human B cells [14] significantly affected the expression of genes, including *EDN1* and *NOS*, known to have important roles in endothelial cell function. We sought to investigate whether *NKX2-3* knockdown would affect important genes and signaling pathways in HIMEC.

*EDN1* was found to be up-regulated by *NKX2-3* knockdown in both HIMEC (array ratio 1.2/1.3, *p*<0.05). *NKX2-3* knockdown significantly affected genes within the endothelin-1 pathway (*p* = 1.02×10^−4^), which was among the top 25 pathways affected by *NKX2-3* (Fig. 2A). Fig. 2B illustrates the genes in the endothelin-1 pathway affected by *NKX2-3* knockdown. *NKX2-3* knockdown activated MAPK through EDN1-PLCβ-PLC pathway, thus regulated inflammation response and cell proliferation.

Nitric oxide (NO) which is generated by NO synthase (NOS) may play an important role in the pathogenesis of IBD [15]. Production of NO (in macrophages) is among the top 10 pathways affected by *NKX2-3* (Fig. 2A). *eNOS* is down-regulated by *NKX2-3* knockdown in both HIMEC (array ratio −1.3/−2.7, *p*<0.0001). IPA analysis showed that the regulation of eNOS can be through VEGF-PI3K/AKT pathway and that this pathway is significantly affected by *NKX2-3* knockdown (*p* = 5.45×10^−3^) (Fig. 2C). Fold changes of key genes in this pathway regulated by *NKX2-3* knockdown in HIMEC are: *VEGF* (−1.3/−1.3), *PI3K* (−1.5/−1.7), and *AKT* (−1.35/−1.35).

### Validation of cDNA microarray by RT-PCR

**Figure 3:**
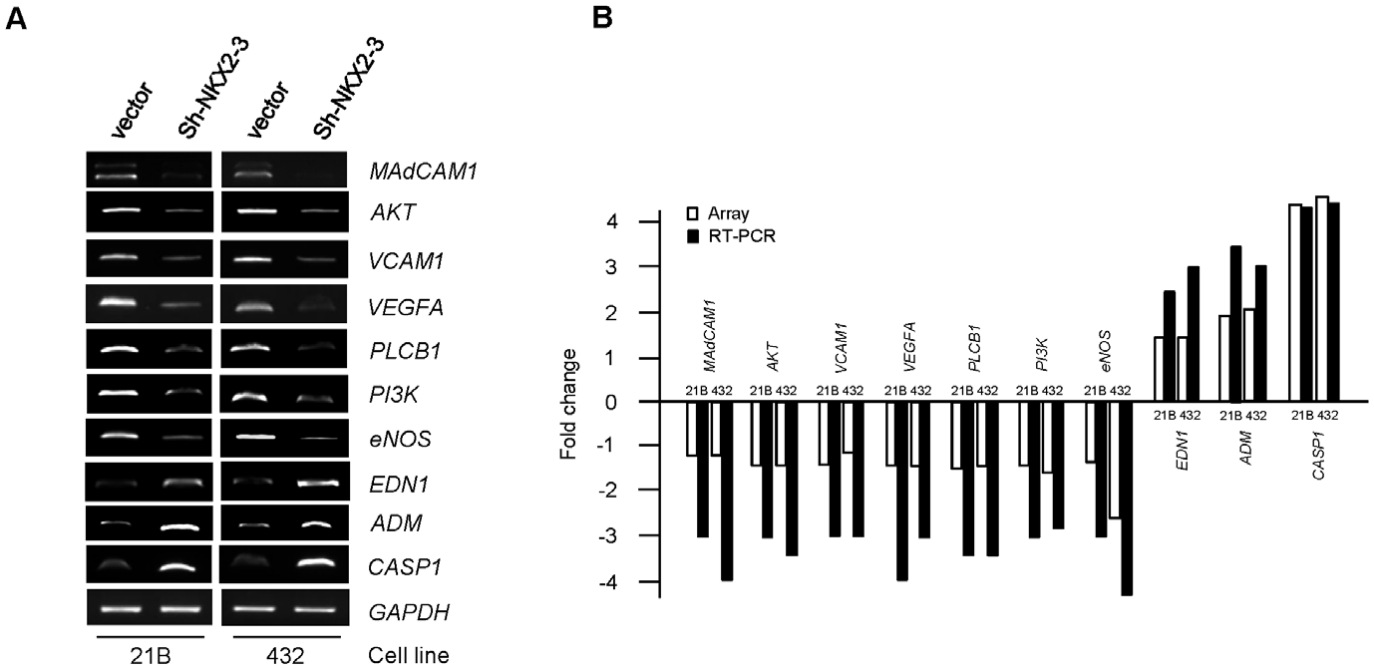
Validation of microarray results by RT-PCR in two *NKX2-3* knockdown HIMEC. (A) RT-PCR analysis of mRNA expression levels of 10 genes in knockdown and control cells. *EDN1, ADM, and CASP1* showed increased mRNA expression levels in *NKX2-3* knockdown cells compared with control cells; *MAdCAM1, AKT, VCAM1, VEGF, PLCB1, PI3K, and eNOS* showed decreased mRNA expression levels in *NKX2-3* knockdown cells compared with control cells. *GAPDH* expression served as a control. (B) Microarray results and corresponding RT-PCR for the 10 genes. The RT-PCR product bands on the photograph were scanned by densitometry. The relative mRNA expression level was expressed as gene expression levels in knockdown cells compared to gene expression levels in control cells.

RT-PCR on 10 genes (involved in or affected by EDN1 and VEGF pathways) was carried out as an independent verification method of the microarray results. Among the 10 genes, seven (*MAdCAM1, AKT, VCAM1, VEGF, PLCB1, PI3K, and eNOS*) were down-regulated by *NKX2-3* knockdown in both HIMEC, and three (*EDN1*, *ADM, and CASP1*) were up-regulated by *NKX2-3* knockdown in 2 HIMEC detected by cDNA microarray. All RT-PCR results were consistent with the microarray data. The fold changes in microarray between the 2 *NKX2-3* knockdown cell lines and 2 control HIMEC lines were: *MAdCAM1* (−1.2;−1.2), *AKT* (−1.35;−1.35), *VCAM1* (−1.3;−1.14), *VEGFA* (−1.3;−1.3), *PLCB1* (−1.6;−1.5), *PI3K* (−1.5;−1.7), *eNOS* (−1.3;−2.7), *EDN1* (1.2;1.3), *ADM* (1.9;2.1), and *CASP1* (4.6;5). The PCR ratios between the 2 *NKX2-3* knockdown cell lines and 2 control HIMEC lines were: *MAdCAM1* (−3;−4), *AKT* (−3;−3.5), *VCAM1* (−3;−3), *VEGFA* (−4;−3), *PLCB1* (−3.5;−3.5), *PI3K* (−3;−2.8), *eNOS* (−3;−4.5), *EDN1* (2.5;3), *ADM* (3.5;3), and *CASP1* (4.5;4.8) (Fig. 3).

### Expression of *NKX2-3*, *EDN1*, *VEGFA*, *PI3K*, *AKT*, and *eNOS* in intestinal tissues from IBD patients

*NKX2-3* is reported to be up-regulated in intestinal tissues in CD patients [13] and VEGF showed markedly enhanced expression levels in both CD and UC tissues [16], while *EDN1* showed both increased levels [17] and decreased levels in intestinal tissues from IBD patients [18]. Since *NKX2-3* can affect the endothelin-1 and VEGF-PI3K/AKT-eNOS pathways (Fig. 2B and C) in HIMEC, we examined mRNA expression levels of the 6 genes in diseased and adjacent normal intestinal tissues from IBD patients to study clinical implication of *NKX2-3* and its regulated genes. 31 CD and 32 UC patients are for this study (Table 2).

**Table 2:** Patient demographic data.

	*Crohn's disease*	*Ulcerative colitis*
	*(n = 31)*	*(n = 32)*
**Sex M/F**	14/17	16/16
**Family history for IBD (yes/no)**	8/23	5/27
**Average age**	39.9±15.1	48.4±16.3
**IBD location**		
terminal ileum	16	1
small bowel	5	1
colon	8	26
cecum	2	3
rectum	0	1

**Figure 4:**
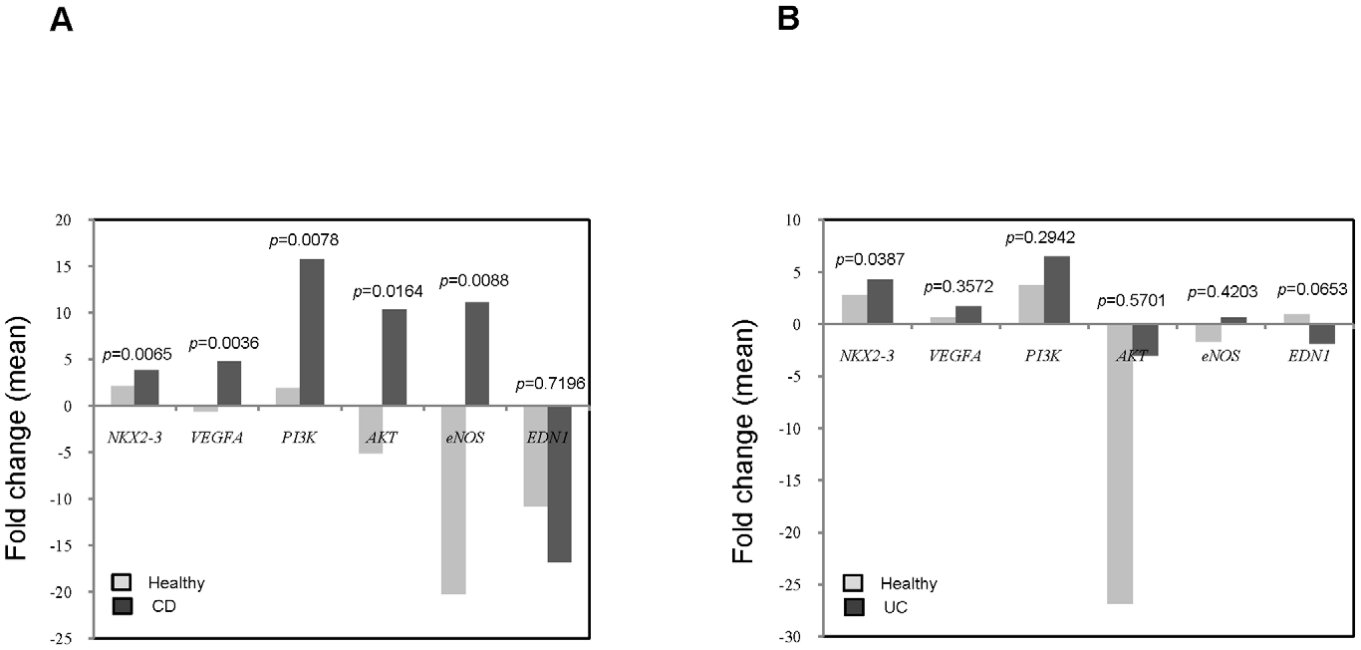
mRNA expression levels of 6 genes in intestinal tissues from CD and UC patients. (A) Comparison of mRNA expression levels of 6 genes in diseased vs. adjacent normal intestinal tissues from CD patients: *NKX2-3* (n = 30), *VEGFA* (n = 30), *PI3K* (n = 31), *AKT* (n = 28), *eNOS* (n = 24), and *EDN1* (n = 30). RT-PCR was carried out on surgically excised diseased and adjacent normal intestinal tissues from CD patients. The RT-PCR product bands on the photograph were scanned by densitometry. RT-PCR results were normalized by *GAPDH* as fold change for each patient. Data are presented as the means. (B) Comparison of mRNA expression levels of 6 genes in diseased vs. adjacent normal intestinal tissues from UC patients: *NKX2-3* (n = 30), *VEGFA* (n = 32), *PI3K* (n = 32), *AKT* (n = 21), *eNOS* (n = 23), and *EDN1* (n = 27). RT-PCR was carried out on surgically excised diseased and adjacent normal intestinal tissues from UC patients. Data are presented as the means.

As shown in Fig. 4A, the mRNA expression levels of *NKX2-3*, *VEGFA, PI3K, AKT, and eNOS* are significantly increased in diseased intestinal tissues compared with adjacent normal tissues in CD patients. The overall mean *NKX2-3* expressions were: 3.88±2.42 (CD) vs. 2.11±1.4 (normal) (*p* = 0.0065); *VEGFA* expressions were: 4.8±15.57(CD) vs. −0.55±7.43 (normal) (*p* = 0.0036); *PI3K* expressions were: 15.78±35.76 (CD) vs. 1.91±7.61 (normal) (*p* = 0.0078); *AKT* expressions were: 10.32±30.75 (CD) vs. −5.05±24.58 (normal) (*p* = 0.0164); and *eNOS* expressions were: 11.13±48.31(CD) vs. −20.22±55.52 (normal) (*p* = 0.0088). Expression levels of *EDN1* are decreased in diseased intestinal tissues compared with adjacent normal tissues in CD patients: −16.82±83.13 (CD) vs. −10.77±26.94 (normal) (*p* = 0.7196).

As shown in Fig. 4B, the mRNA expression levels of *NKX2-3*, *VEGFA, PI3K, AKT, and eNOS* are increased in diseased intestinal tissues compared with adjacent normal tissues in UC patients. The overall mean *NKX2-3* expressions were: 4.26±3.39 (UC) vs. 2.78±1.68 (normal) (*p* = 0.0387); *VEGFA* expressions were: 1.7±1.53 (UC) vs. 0.65±3.13 (normal) (*p* = 0.3572); *PI3K* expressions were: 6.46±7.63 (UC) vs. 3.73±5.22 (normal) (*p* = 0.2942); *AKT* expressions were: −3±5.63 (UC) vs. −26.79±45.65 (normal) (*p* = 0.5701); and *eNOS* expressions were: 0.62±2.04 (UC) vs. −1.67±5.19 (normal) (*p* = 0.4203). Expression levels of *EDN1* are decreased in diseased intestinal tissues compared with adjacent normal tissues in UC patients: −1.85±8.7 (UC) vs. 0.97±4.29 (normal) (*p* = 0.0653).

Taken together, expression levels of *NKX2-3*, *VEGFA*, *PI3K*, *AKT*, and *eNOS* are increased in intestinal tissues from IBD patients. On the other hand, expression levels of *EDN1* are decreased in intestinal tissues from IBD patients. These results demonstrated the important roles of these genes in IBD pathogenesis.

### Positive correlation of gene expression of *NKX2-3* with *VEGFA* in intestinal tissues from IBD patients

**Figure 5:**
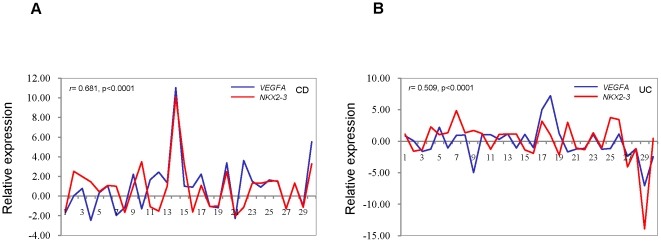
The correlation between the expression levels of *NKX2-3* and *VEGFA* in intestinal tissues from IBD patients. (A) The correlation between the expression levels of *NKX2-3* and *VEGFA* in diseased and adjacent normal intestinal tissues from 30 CD patients. RT-PCR was carried out on surgically excised intestinal tissues. The RT-PCR product bands on the photograph were scanned by densitometry. RT-PCR results were normalized by *GAPDH* for each sample. Fold change results of normalized *NKX2-3* or *VEGFA* were summarized as a ratio of medians (CD: adjacent normal tissue) in every patient. Correlation coefficient (*r*) and *p* value are shown. (B) The correlation between the expression levels of *NKX2-3* and *VEGFA* in diseased and adjacent normal intestinal tissues from 30 UC patients.

There are seven members of the vascular endothelial growth factors (VEGFs) family, ie, VEGF-A, -B, -C, -D, -E, -F, and placental growth factor. *VEGFA* is crucially involved in several chronic inflammatory disorders in which it not only promotes pathologic angiogenesis but directly fosters inflammation [19], [20]. VEGFA has been reported to over-express in humans with IBD [21], [22], [23]. *VEGFA* was down-regulated by *NKX2-3* knockdown in 2 HIMEC (Fig. 2C and 3). Since both *NKX2-3* and *VEGFA* are up-regulated in intestinal tissues from IBD patients (Fig. 4), next we examined whether *NKX2-3* expression was correlated with *VEGFA* expression in IBD patients. We examined mRNA expression levels of *NKX2-3* and *VEGFA* in diseased and adjacent normal intestinal tissues from 30 CD and 30 UC patients. Fold change results of normalized *NKX2-3* or *VEGFA* were summarized as a ratio of medians (CD or UC: adjacent normal tissue) in every patient. Correlation analysis showed a positive correlation between expression of *NKX2-3* and *VEGFA* (*r* = 0.681, *p*<0.0001) for CD (Fig. 5A) and for UC (*r* = 0.509, *p*<0.0001) (Fig. 5B).

### Negative correlation of gene expression of *NKX2-3* with *EDN1* in intestinal tissues from IBD patients

**Figure 6:**
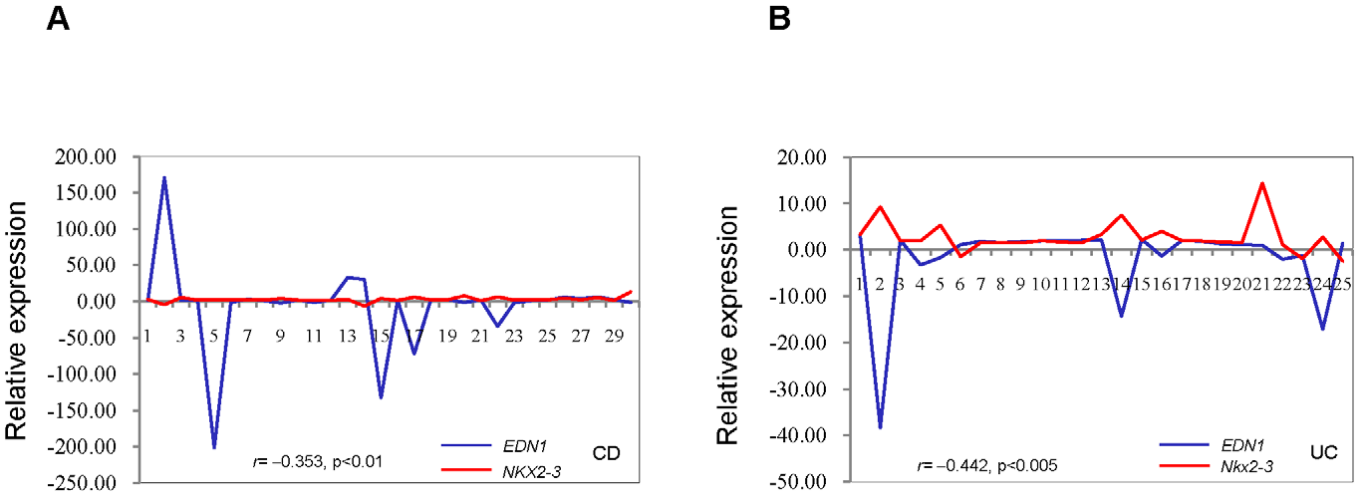
The correlation between the expression levels of *NKX2-3* and *EDN1* in intestinal tissues from IBD patients. (A) The correlation between the expression levels of *NKX2-3* and *EDN1* in diseased intestinal tissues from 30 CD patients. RT-PCR was carried out on surgically excised intestinal tissues. The RT-PCR product bands on the photograph were scanned by densitometry. RT-PCR results were normalized by *GAPDH* in every patient. Correlation coefficient (*r*) and *p* value are shown. (B) The correlation between the expression levels of *NKX2-3* and *EDN1* in diseased intestinal tissues from 25 UC patients.

Endothelin-1 (*EDN1*) is a vasoactive peptide implicated in a number of pathological conditions, including human IBD [17]. *EDN1* was up-regulated by *NKX2-3* knockdown in 2 HIMEC by cDNA microarray analysis (Fig. 2B) and this observation was confirmed by RT-PCR (Fig. 3). Since *NKX2-3* is up-regulated and *EDN1* is down-regulated in intestinal tissues from IBD patients (Fig. 4), we examined whether *NKX2-3* expression was correlated with *EDN1* expression in IBD patients. We examined mRNA expression levels of *NKX2-3* and *EDN1* in diseased intestinal tissues from 30 CD and 25 UC patients. Correlation analysis showed a negative correlation between expression of *NKX2-3* and *EDN1* for CD (*r* = −0.353, *p*<0.01) (Fig. 6A) and for UC (*r* = −0.442, *p*<0.005) (Fig. 6B).

## Discussion

Pathogenesis of IBD is not only restricted to those mediated by classic immune cells, such as T and B cells [24], but also involves nonimmune cells, including endothelial cells [15]. Endothelial cells play a key role in mucosal immune homeostasis by regulating the leukocytes migrating from the intravascular to the interstitial space, thus highlighting the endothelium as one of the pillars in inflammation pathogenesis [25]. The vascular response is a key component of inflammation. Microvascular endothelial cells could form capillary-like structures and display different functional sets of adhesion molecules, distinct chemokine secretory patterns, and activation of unique sets of genes in response to stress and inflammatory stimuli [26].

In this study, we performed genome-wide gene expression microarray analysis using two HIMEC with *NKX2-3* knockdown, and identified 1746 genes in 21B cells and 1603 genes in 432 cells regulated by *NKX2-3* knockdown. The regulation of these genes is highly consistent between the two HIMEC. Most of the *NKX2-3* regulated genes are involved in immune and inflammatory response, cell proliferation and growth, metabolic process, and angiogenesis. Various aspects of immunity contribute to the development of an overall inflammatory immune response. Chemokines control leukocyte trafficking during homeostasis as well as inflammation. The migration of leukocytes into sites of inflammation is crucial in the pathogenesis of IBD. The CXC chemokine IL8/CXCL8 is down-regulated by *NKX2-3* knockdown in HIMEC. IL8 is expressed in leucocytes and endothelial cells and plays an important role in inflammation and angiogenesis [27]. The expression level of IL8 is up-regulated in tissues after ischaemic injury [28]. One of the most novel aspects that directly implicates endothelial cell participation in inflammation is the process of angiogenesis. It is now well established that angiogenesis and microvascular remodeling are intrinsic components of the tissue remodeling in chronic inflammatory diseases [15]. ANGPT2 (angiopoietin 2) is down-regulated by *NKX2-3* knockdown in HIMEC. ANGPT2 is responsible for the initiation of angiogenesis through recruitment and proliferation of endothelial cells [29]. Elevated serum ANGPT2 levels in IBD patients have been reported [30].

We used pathway analysis to systematically visualize the relationships of genes regulated by *NKX2-3* knockdown. The top 25 pathways affected by *NKX2-3* knockdown included those involved in inflammation and immune response, including the EDN1 and NO pathways. Generation of nitric oxide (NO) by NO synthase (NOS) is a central feature of chronic inflammatory diseases in the gastrointestinal tract [15]. *eNOS* is down-regulated by *NKX2-3* knockdown in HIMEC. We identified that *NKX2-3* regulated *eNOS* through the VEGF-PI3k/AKT pathway in HIMEC. *VEGF, PI3K, and AKT* are all down-regulated by *NKX2-3* knockdown in HIMEC. A few reports have described overexpression of VEGFA in human intestinal tissues with IBD [21], [22], [23], but the functional significance of such up-regulation is not yet understood. In murine colonic-derived endothelial cells, VEGFA triggers an inflammatory phenotype by up-regulating CAMs and inducing adhesion of neutrophils and T cells, thus supporting an inflammatory role for this cytokine in the intestine [31]. One recent study showed that in vitro, VEGF-A induces both angiogenic activity and an inflammatory phenotype in human intestinal microvascular endothelial cells (HIMEC), whereas overexpression in vivo increases disease severity and blockade decreases disease severity in colitic mice [23]. Given that the activation of this VEGF–eNOS angiogenesis pathway results in maintenance production of NO, it is most likely to be implicated in the regulation of NO-controlled gene expression, as well as the NO-mediated angiogenic responses to VEGF. VEGF is known to be a potent activator of endothelial cells and a critical factor for the induction of angiogenesis during IBD development [23]. The importance of the VEGF–eNOS pathway for regulating angiogenesis and vascular remodeling makes these genes important for IBD pathogenesis. Our results showed that mRNA expression levels of *NKX2-3*, *VEGFA, PI3K, AKT, and eNOS* were increased in intestinal tissues from IBD patients. And the positive correlation between the expression levels of *NKX2-3* and *VEGFA* in intestinal tissues from IBD patients (Fig. 5) suggests a regulatory role of *NKX2-3* in *VEGFA* gene expression. The mechanism of regulation of *VEGF* by *NKX2-3* is not clear. *NKX2-3* could regulate expression of *IL8, ANGPT2, KLF2, and KLF4* in HIMEC. IL8 Stimulates VEGF expression in endothelial cells [32] and ANGPT2 stimulates the synthesis of VEGF [33]. The KLF2 and KLF4 transcription factors have been shown to coordinate transcriptional programs important for the establishment of an anti-inflammatory, vasodilatory, and anti-thrombotic vascular endothelial phenotypes, thus acting as critical regulators of endothelial homeostasis [34]. KLF2 and KLF4 regulate expression of VEGF and eNOS in vascular endothelial cells [34]. It is reasonable to speculate that *NKX2-3* regulates *VEGF* expression through *IL8, ANGPT2, KLF2, and KLF4* or direct regulation of *VEGF*. Although the role of *NKX2-3* in IBD is still unclear, its regulatory role in VEGF-eNOS strongly suggests that *NKX2-3* could be involved in IBD pathogenesis by regulating the VEGF-PI3K/AKT-eNOS pathway.

Endothelin-1 (*EDN1*) is one of several vasoconstrictors that may play a role in the progression of IBD. Endothelin-1 is expressed by endothelial cells as a precursor peptide (proET-1) that is first cleaved to bigET-1 and then to the mature 21-amino acid peptide. *EDN1* acts through two receptors, ET-A and ET-B. EDN1 binding to ET-B receptors in endothelial cells initiates a signaling cascade that leads to nitric oxide (NO) and endothelial-derived relaxing factor production. The signaling of NO production through ET-B receptors via the G-protein βγ subunit dimer has been linked to AKT phosphorylation of eNOS. The G-protein Galpha12 has been linked to increased levels of eNOS [35]. EDN1 increases leukocyte adhesion to intestinal microvasculature [36], resulting in oxidant stress and mucosal dysfunction [37]. Increased levels of EDN1 in intestinal tissues from IBD patients have been reported [17]. However, other reports have indicated that EDN1 levels were decreased in IBD [18], [38]. We showed that expression of EDN1 was down-regulated in intestinal tissues from IBD patients. Correlation study confirmed the negative correlation between the expression levels of *NKX2-3* and *EDN1* in intestinal tissues from IBD patients (Fig. 6), suggesting a regulatory role of *NKX2-3* in *EDN1* gene expression. *NKX2-3* could negatively regulate *EDN1* expression and is thus involved in IBD pathogenesis.

In summary, this work identified many inflammation-related genes and microvascular endothelial cell function related genes that are regulated by *NKX2-3* in HIMEC. Our present results demonstrate that a decrease in *NKX2-3* gene expression level can profoundly affect the signaling pathways relevant to the pathogenesis and progression of IBD such as the EDN1 and VEGF signaling and PI3K/AKT-eNOS pathways. Further functional studies of the genes and pathways affected by *NKX2-3* using HIMEC are underway to confirm and expand upon the current work.

## Materials and Methods

### Cell lines

Two HIMEC (432 and 21B) were isolated from normal ileal intestinal tissue from patients undergoing surgery at the Cleveland Clinic Lerner Research Institute as approved by the Institutional Review Board of University Hospitals of Cleveland. Cell lines were cultured in MCDB131 medium (Invitrogen, USA) supplemented with 20% fetal bovine serum (FBS), heparin, endothelial cell growth factor and antibiotics. HIMEC were incubated at 37°C in an atmosphere of 5% CO_2_.

### Transfection

A small hairpin RNA (shRNA) vector targeting human *NKX2-3* was generated using the pSUPER vector system as described previously [14], [39]. The 19-nucleotide sequence within *NKX2-3* targeted by the shRNA oligonucleotide pair was 5′-AGGAACATGAAGAGGAGCC-3′. Forward and reverse primers were synthesized containing this sequence in sense and antisense orientations with an intervening linker. Forward and reverse primers were annealed and ligated into the pSUPER.retro.puro vector according to the manufacturer's instructions (OligoEngine, WA, USA).

pSUPER.retro.puro.shRNA-*NKX2-3* and empty vector were transfected into two HIMEC lines with TransPass™ HUVEC transfection reagent (New England BioLabs, USA) according to the manufacturer's instructions. Forty-eight hours posttransfection, to confirm the suppression of *NKX2-3* expression, mRNA from was isolated from the transfected HIMEC lines for further examination.

### Microarray and data analysis

Illumina Human HT12 v 3 Expression BeadChips (Illumina, CA) were used in this study. This BeadChip targets >25,000 genes with >48,000 probes derived from the RefSeq (Build 36.2, rel22) and UniGene (Build 99) databases. 300 ng of total RNA collected from two independent cultures of control and sh*NKX2-3*-expressing HIMEC cells was reverse transcribed into cRNA and biotin-UTP labeled using the Illumina Total Prep RNA Amplification Kit (Ambion, Austin, TX). Microarray hybridization, data collection, and analysis were performed at the Genomics Core of the Cleveland Clinic Lerner Research Institute.

Raw data filtering and quantile normalization were performed using the Bioconductor package *lumi*, a Beadarray specific software package for Illumina microarray data. Due to the small sample size, the moderated t-statistic implemented in the Bioconductor *LIMMA* package was used to detect differentially expressed genes. This statistic has the same interpretation as the standard t-statistic; however, standard errors were calculated to shrink toward a common value by empirical Bayes model to borrow information across all genes [40]. The *p*-values from moderated t-tests were adjusted by Benjamini and Hochberg's method to control false discovery rate. All data is MIAME (Minimum Information About a Microarray Experiment) compliant and that the raw data has been deposited in a MIAME compliant database (GEO at NCBI, accession number GSE28656).

### Ingenuity pathway analysis (IPA)

IPA was used to identify gene networks according to biological functions and/or diseases in the Ingenuity Pathways Knowledge Base (Ingenuity Systems, Redwood City, CA). Known genes' expression levels served as input to the Ingenuity Pathways Analysis (IPA) Knowledge Base v4.0. Lists of top pathways associated with genes with changes in expression relative to controls were generated with corresponding Benjamini and Hochberg's p values. Expression data was overlaid upon canonical pathways associated with altered gene expression.

### Patients and intestinal tissue samples

Intestinal tissues were obtained from IBD patients undergoing surgery at the Penn State Hershey Medical Center. All human tissues were approved by the Human Subjects Protection Offices of The Pennsylvania State University College of Medicine, and were undertaken with the understanding and written consent of each subject. Macroscopically normal areas of intestine and areas of intestine with obvious disease were classified by a pathologist. The intestinal tissues were immediately submerged in RNA*later* Solution (Ambion, CA, USA) and stored at 4°C overnight. Tissues were stored frozen at −70°C until total RNA extraction.

### RNA isolation and reverse transcription PCR

Total RNA was extracted from HIMEC and intestinal tissues using the RNeasy mini kit (QIAGEN Sciences, MD, USA) according to the manufacturer's instructions. cDNA was synthesized from 1.0 µg of total RNA using a Superscript III 1^st^ Strand Synthesis Kit (Invitrogen, CA, USA).

Primer sequences are listed in table 3. Primers were designed using Primer3 software. PCR amplifications were performed at 94°C for 30 seconds, 60°C for 45 seconds, and 72°C for 45 seconds for 35 cycles (all genes except GAPDH) or 26 cycles (GAPDH), followed by a final extension at 72°C for 5 min. PCR products were visualized on 2% agarose gels stained with ethidium bromide. RT-PCR product bands were scanned by densitometry and results were normalized by GAPDH for each cell line.

**Table 3:** Primers for RT-PCR.

Gene	Primer sequences	Sizes	Accession
EDN1	F: 5′- ctttgagggacctgaagctg-3′	392 bp	NM_001955
	R: 5′- ctgttgcctttgtgggaagt-3′		
ADM	F: 5′- acttcggagttttgccattg-3′	230 bp	NM_001124
	R: 5′- ctcttcccacgactcagagc-3′		
PI3K	F: 5′- tgctttgggacaaccataca-3′	394 bp	NM_006218
	R: 5′- cggttgcctactggttcaat-3′		
AKT	F: 5′- ggtgatcctggtgaaggaga-3′	379 bp	NM_005163
	R: 5′- cttaatgtgcccgtccttgt-3′		
eNOS	F: 5′- tgctggcatacaggactaag-3′	385 bp	NM_000603
	R: 5′- taggtcttggggttgtcagg-3′		
PLCB1	F: 5′- cgtggctttccaagaagaag-3′	305 bp	NM_015192
	R: 5′- ggcaaaggttgttgaggaaa-3′		
CASP1	F: 5′- acctctgacagcacgttcct-3′	329 bp	NM_033292
	R: 5′- ggtgtggaagagcagaaagc-3′		
VCAM1	F: 5′- attgacttgcagcaccacag-3′	391 bp	NM_001078
	R: 5′- ttccagggacttcctgtctg-3′		
VEGFA	F: 5′- tcctcacaccattgaaacca-3	379 bp	NM_001171623
	R: 5′- caccgatcagggagagagag-3′		
NKX2-3	F: 5′-ccacccctttctcagtcaaa-3′	210 bp	NM_145285
	R: 5′-ctgcggctagtgagttcaaa-3′		
GAPDH	F: 5′-tgatgacatcaagaaggtggtgaag-3′	236 bp	NM_002046
	R: 5′-tccttggaggccatgtgggccat-3′		

### Statistical analysis

The paired data (RT-PCR results) were analyzed with a one sample permutation t-test with 500,000 permutations. The analysis was performed using R (version 2.12.0) and the onet.permutation function in the R library DAAG (version 1.03).
